# Three-Dimensional Pulse-Based Modelling of Femtosecond Laser Ablation of Metals: Validation with Grooves

**DOI:** 10.3390/mi14030593

**Published:** 2023-03-01

**Authors:** Pol Vanwersch, Balasubramanian Nagarajan, Albert Van Bael, Sylvie Castagne

**Affiliations:** 1 KU Leuven, Department of Mechanical Engineering and Flanders Make@KU Leuven - M&A, Celestijnenlaan 300, B-3001 Leuven, Belgium; 2 KU Leuven, Department of Materials Engineering, Diepenbeek Campus, Wetenschapspark 27, B-3590 Diepenbeek, Belgium

**Keywords:** femtosecond laser modelling, ultrafast laser ablation, stainless steel

## Abstract

The femtosecond (fs) laser ablation of metals is a precise method used to create microfeatures on the surface of the material with a minimized heat-affected zone (HAZ). Despite its many advantages, fs laser ablation often requires extensive trial-and-error experimentation before finding the optimal laser strategy for a desired geometry with minimal HAZ. The pulse-based two-temperature model (TTM) can significantly shorten this process by predicting the ablated geometry based on a set of material and laser parameters. However, this model has only been validated for percussion drilling and single lines. In this study, the pulse-based TTM is tested against parallel line experiments and subsequently modified to include geometry-dependent material parameters. More specifically, the threshold fluence and reflectivity of the material are modified to incorporate the temperature increase inside the standing features between parallel lines. The introduced geometry-dependent factors are fitted with experimental data and their inclusion in the model is shown to have a positive impact on the simulation results. The results show a clear amelioration in the shape and depth of the simulated profiles, with the error on the average depth and width of the modified TTM being lower than the average standard deviation on the experiments.

## 1. Introduction

Femtosecond (fs) laser ablation, also referred to as ultrashort pulse laser ablation, is a highly precise and controllable process used for micromachining and surface texturing applications [1]. The short laser pulse (~100s of femtoseconds) delivers energy to the electrons in the target material while, during a few picoseconds or less, the lattice remains at a lower temperature. The energy is only transferred from the electrons to the lattice after a certain time (longer than the pulse duration), depending on the electron–phonon relaxation time. fs laser ablation is typically modelled by the two-temperature model (TTM), describing the spatial and temporal evolution of the temperature of electrons and the lattice in the target material following an ultrashort pulse [2]:

(1) $$C_{e}\frac{\partial T_{e}}{\partial t}=-\frac{\partial Q\left(k\right)}{\partial k}-\gamma \left(T_{e}-T_{l}\right)+S$$

(2) $$C_{l}\frac{\partial T_{l}}{\partial t}=\gamma \left(T_{e}-T_{l}\right)$$

where the subscripts *e* and *l* refer to the electron and lattice parameters, respectively; *T* is the temperature; *C* is the heat capacity; *k* is the direction perpendicular to the target surface; *Q*(*k*) is the heat flux; *γ* is the parameter characterizing the electron–lattice coupling; and *S* represents the laser heating source. Based on the above equations, and assuming ablation when $C_{l}T_{l}$ reaches a certain threshold, the ablation depth per pulse $\left(z\right)$ can be calculated as follows: [2]

(3) $$z=max\{\delta \times ln(\frac{F_{a}}{F_{th}}),0\}$$

where *δ* is the optical penetration depth of electromagnetic radiation in the target material, which is also defined as the inverse of the absorption coefficient *α*. $F_{a}$ is the absorbed laser fluence and $F_{th}$ is the ablation threshold fluence, meaning that ablation will occur when $F_{a}>F_{th}$. Contrary to Equations (1) and (2), where the spatial and temporal evolution of the electron and lattice temperatures are calculated following each pulse, the pulse-based TTM (Equation (3)) focuses on the end state of the material following fs laser ablation, without having to determine the state of the irradiated material at every time step following a pulse [2]. In our previous work [3], a method for calculating the absorbed fluence was developed and validated for percussion drilling with a Gaussian beam:

(4) $$F_{a}\;\left(x,y\right)=F_{c}\times e^{-\left(\frac{2\left({\left(x-x_{c}\;\right)}^{2}+{\left(y-y_{c}\;\right)}^{2}\right)}{\omega_{0}^{2}}\right)}\times \frac{A_{proj}}{A_{real}}\times \left(1-R\right)$$

where $x_{c}$ and $y_{c}$ are the coordinates of the pulse center, $\omega_{0}$ is the beam focus radius at 1/e² of the peak fluence, R is the reflectivity, and $A_{proj}/A_{real}$ is the area correction factor. The area correction factor allows the inclination of the local surface to be taken into account in a three-dimensional environment. $A_{real}$ is the actual surface area represented by mesh point (*x*, *y*), and $A_{proj}$ is the projected surface area represented by the same point on a plane perpendicular to the propagation direction of the laser beam. $F_{c}$ is the peak laser fluence and can be expressed in the function of the delivered pulse energy $E_{P}$ as follows:

(5) $$F_{c}=2\times \frac{E_{P}}{\pi \times \omega_{0}^{2}}$$

For deep features, it is important to take into account the beam divergence. Therefore, $\omega_{0}$ from Equations (4) and (5) can be rewritten in function of the depth z:

(6) $$\omega_{0}\left(z\right)=\omega_{0}\times \sqrt{1+{\left(\frac{z-z_{focus}}{z_{R}}\right)}^{2}}$$

where $z-z_{focus}$ is the difference between the height of the mesh point and the laser focus height, and $z_{R}$ is the Rayleigh length of the beam.

It is commonly accepted that, for a low number of pulses (N<20), the threshold fluence and the penetration depth decrease with every pulse [4,5]. This is called the incubation effect, which is caused by energy absorbed by the target material during the previous pulses. The pulse-dependent threshold fluence and penetration depth can be described by the following equations [6,7]:

(7) $$F_{th}\left(N\right)=F_{th}\left(1\right)\times N^{S-1}$$

(8) $$\delta \left(N\right)=\delta \left(1\right)\times N^{S-1}$$

where N is the number of delivered pulses and S is the incubation factor (typically 0.8 for metals [8]). However, the decrease in the penetration depth with an increasing number of pulses is often ignored and replaced by a constant penetration depth in the literature. For a higher number of pulses (N>20), $F_{th}$ and δ are constant and equal to $F_{th}\left(20\right)$ and $\delta \left(20\right)$, respectively [9].

While these relationships provide accurate results for percussion drilling [3,4] and single line scanning [10], they are yet to be validated for parallel line scanning or even more complicated scanning strategies, where the effect of accumulated heat plays a significant role.

## 2. Temperature Influence in Parallel Line Scanning 

In parallel line scanning, the hatch pitch (i.e., the distance between two lines) is, in general, considerably larger than the pulse pitch (i.e., the distance between two pulses in one line). As a result, interactions between lines cannot be considered in the same way as the interactions between pulses within a line. 

For pulses within one line, the repetition rate is typically high (in the order of hundreds of kHz) and the pulse pitch is low (in the order of a few µm). Hence, the target material, which is heated up by part of the fluence under the ablation threshold, does not cool down completely before the deposition of the next pulse. This phenomenon is typically represented by the incubation factor (Equations (7) and (8)). 

**Figure 1:**
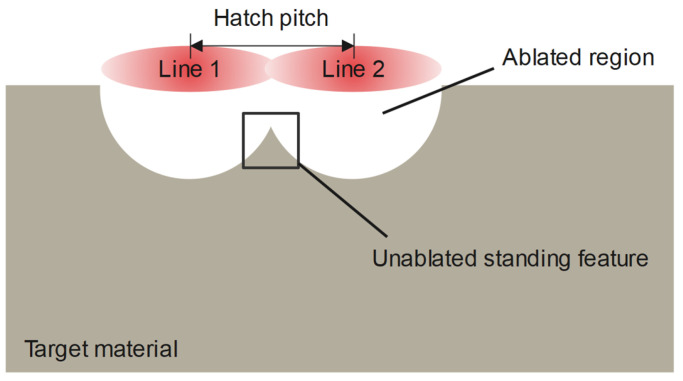
Illustration of the unablated standing feature between parallel lines with a large hatch pitch.

The time between the scanning of parallel lines is considerably longer than the time between the consecutive pulses in one line. Therefore, the material can be considered to have cooled down to the room temperature before the ablation of the next line and the incubation can be reset before the simulation of each line. However, the larger hatch pitch means that an unablated standing feature can be present between the parallel lines during ablation. An illustration of this phenomenon is shown in Figure 1. 

The temperature in this feature is typically higher than what is considered by the incubation factor because of the larger surface-to-volume ratio. In this study, the above-mentioned model is updated to include temperature-dependent factors in order to improve its accuracy for groove-like features. Subsequently, it is compared to, and validated with, experimental data.

Heinigk et al. [11] showed that the threshold fluence dependence on the temperature can be expressed as follows:

(9) $$F_{th}\left(T\right)=F_{th}\left(T_{0}\right)-\rho C_{p}\left(T-T_{0}\right)d_{heat}$$

where $T_{0}$ is the temperature at which $F_{th}$ is (experimentally) determined, $u=\rho C_{p}\left(T-T_{0}\right)$ is the internal energy, and $d_{heat}$ is the stored heat. Equation (9) can be rewritten as follows:

(10) $$F_{th}\left(T\right)=F_{th}\left(T_{0}\right)-c_{1}\left(T-T_{0}\right)$$

where $c_{1}=\rho C_{p}d_{heat}$ is a material-dependent constant.

The temperature also has an influence on the reflectivity of metals. The reflectivity R is dependent on the electrical conductivity σ [12]:

(11) $$R=1-\frac{2\mu \varepsilon_{0}\omega }{\sigma }$$

where μ is the magnetic susceptibility, $\varepsilon_{0}$ is the permittivity of free space, and ω is the radian frequency. The dependence of the electrical conductivity σ on the temperature is also known [13]:

(12) $$\sigma \left(T\right)=\frac{\sigma_{0}}{\left(1+\alpha_{el}\left(T-T_{0}\right)\right)}$$

where $\alpha_{el}$ is the temperature coefficient of electrical resistance. Combining Equations (11) and (12) gives the temperature dependence of the reflectivity:

(13) $$R\left(T\right)=R\left(T_{0}\right)-\frac{2\mu \varepsilon_{0}\omega }{\sigma_{0}}\alpha_{el}\left(T-T_{0}\right)$$

Equation (13) can be rewritten as follows:

(14) $$R\left(T\right)=R\left(T_{0}\right)-c_{2}\left(T-T_{0}\right)$$

where $c_{2}=\frac{2\mu \varepsilon_{0}\omega }{\sigma_{0}}\alpha_{el}$ is the second material-dependent constant.

In this modelling approach, it is assumed that the non-ablated material remains solid during and after the energy deposition. This means that no melt pool is created, and the material is either heated up in the solid state (F < $F_{th}$) or ablated (F > $F_{th}$). 

It is possible to couple the TTM with FEM simulations to calculate the temperature of the material before every pulse in order to define $F_{th}\left(T\right)$ and $R\left(T\right)$ for every point [11]. This can first be achieved by exporting the material geometry to an FEM solver to calculate the heat distribution and then the material parameters can be updated into the TTM, but this approach would significantly increase the modelling time. In order to avoid this calculation-intensive process, this study uses an empirical approach to determine the relationship between the crater geometry and the material temperature. This method requires more assumptions and is less versatile, but allows for faster calculations.

### The Relationship between the Temperature Gradient and the Feature Geometry

In order to find the relationship between the temperature gradient and the feature geometry, the temperature distribution inside a longitudinal feature is calculated after the application of a defined heat flux using the ANSYS transient thermal simulation module (version 2020 R2). During laser irradiation, the ablation is driven by the absorbed fluence greater than the threshold level (F > $F_{th}$), whereas the remaining fluence (F < $F_{th}$) contributes to heating. In these simulations, the heat flux represents only the absorbed laser energy (F < $F_{th}$) which is responsible for heating.

The simulations are performed on stainless steel material using the standard ANSYS database, with a constant heat convection of 20 W/m^2^·°C and an ambient air temperature of 22 °C. Heat loss due to radiation is not considered in this study. The uniformly distributed heat flux is set at 275 MW/cm² on the upward-facing surfaces for a duration of 0.2 ns. This corresponds to a total fluence of 0.055 J/cm² for pulses of 250 fs. The cooling time is set at 2 µs, which corresponds to the time between two pulses at a 500 kHz repetition rate. The variable parameters are feature height and angle, and the temperature is recorded at different heights in the feature (see Table 1). A measurement height of 0 µm corresponds to the widest point of the feature.

**Figure 2:**
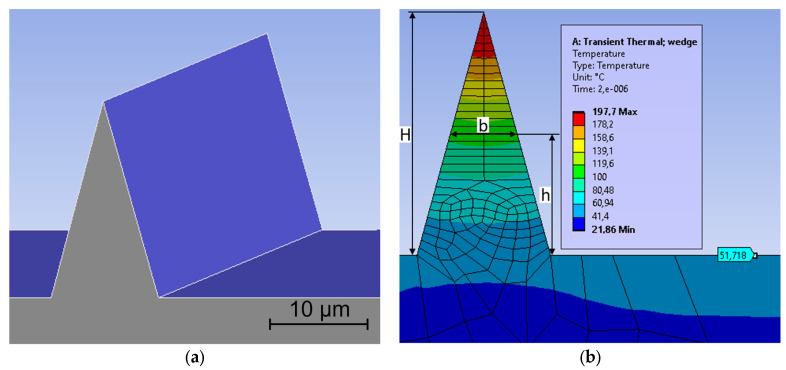
Heat transfer simulations in ANSYS. (**a**) Isometric view of a standing feature. The heat flux is applied on the blue surfaces. (**b**) Temperature profile for a standing feature with a height (H) of 15 µm and a 30° angle. H is the standing feature height, h is the measurement height, and b is the local width at measurement height h.

For these simulations, a cube of material with a 100 µm side is used to represent the bulk material. The standing feature, representing the “Unablated standing feature” inside the ablated crater from Figure 1, is positioned on the top surface of this cube and the heat flux is applied on all upward-facing surfaces, as shown in Figure 2a. The mesh size is set to adaptive with a maximum size of 0.5 µm on the faces of the standing feature, and the time increment step is set to “program controlled” with the initial, minimum, and maximum time steps of 20 ps, 2 ps, and 200 ps, respectively. The temperature profile inside a feature with a 15 µm height and a 30° angle is shown in Figure 2b.

**Figure 3:**
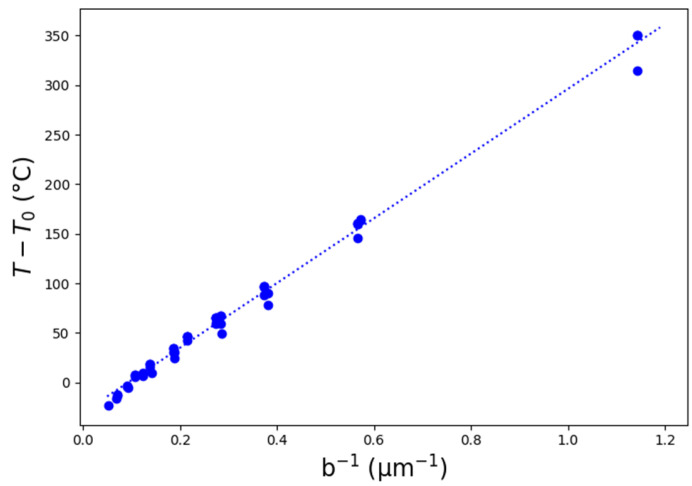
Simulation results of the temperature inside standing features with respect to the local width (b).

In Figure 2b, we see that the temperature increases with increasing height in the standing feature. Additionally, we see that the surface without a standing feature is only slightly heated up by the pulse ($T_{0}$ ≈ 52 °C), which is to be expected. Figure 3 shows the difference between the temperature in the feature and the temperature on the top surface without standing features in the function of the inverse of the local feature width b. Each data point corresponds to a combination of feature height, feature angle, and measurement height (see Table 1). 

Figure 3 demonstrates that the temperature inside the standing feature is inversely proportional to the local width of that feature. This relationship indicates that the influence of convection can be neglected due to the small feature dimensions and the short cooling time. In the current calculations, the heat flux and the cooling time are considered equal for all pulses. This assumes that the absorbed heat density is equal to the threshold fluence for every pulse in the ablation region, and that the repetition rate remains constant throughout the experiments. Because of the observed proportionality, a third constant $c_{3}$ can be introduced based on the heat transfer simulations (Figure 3):

(15) $$T-T_{0}=\frac{c_{3}}{b}$$

where b is the local feature width. By combining Equation (15) with Equations (10) and (14), respectively, the following can be obtained:

(16) $$F_{th}\left(T\right)=F_{th}\left(T_{0}\right)-\frac{c_{Fth}}{b}$$

(17) $$R\left(T\right)=R\left(T_{0}\right)-\frac{c_{R}}{b}$$

where $c_{Fth}=c_{1}c_{3}$ and $c_{R}=c_{2}c_{3}$ are the constants for the threshold fluence correction and the reflectivity correction, respectively. 

## 3. Materials and Methods

### 3.1. Experiments

The target material used for the experiments is a low corrosion tool steel *Stavax* (grade 1.2083;AISI 420, soft annealed, approx. 190 HB). Before laser scanning, the sample is grinded and polished with a 6 µm diamond paste. *Stavax* is assumed to have similar optical and thermal properties as stainless steel AISI 316L, which has been widely studied in the literature. The constants describing the behavior of the material during fs laser ablation are given in Table 2, and have been validated in a previous study for percussion drilling [3]. The important material parameters for the model are the complex refractive index *n* + *κ*i at the 1030 nm wavelength, the penetration depth *δ*, the threshold fluence *F_th_*, and the incubation factor *S*.

**Figure 4:**
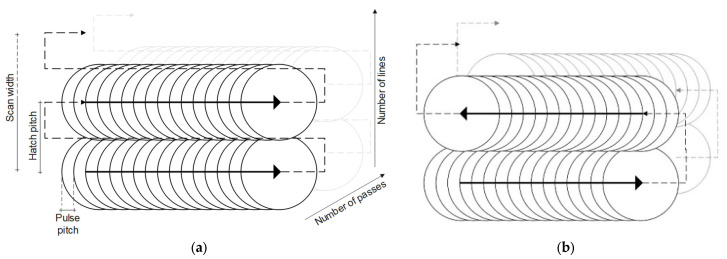
Schematic of the scanning parameters for (**a**) unidirectional and (**b**) bidirectional strategy. The continuous bold arrows represent the laser path in the pulsed mode. The dotted arrows represent the laser path without ablation.

The laser source is a femtosecond laser (SATSUMA) obtained from Amplitude Systèmes (France). The focal length of the focusing lens is 100 mm. The laser generates a beam with a Gaussian profile at the 1030 nm wavelength and circular polarization after the quarter-wave plate. The maximum average power delivered on target is 7.7 W and the pulse length is 250 fs. The beam focus radius ($\omega_{0}$) is 8.2 μm [15]. In this study, the parallel line scanning experiments are performed at normal atmospheric conditions and with a fixed vertical scanner position. After the ablation (and before characterization), the samples are cleaned in an ultrasonic bath for 10 min. An overview of the laser machining parameters is given in Table 3. The varied parameters are the hatch pitch, the number of passes (i.e., the number of repetitions of the same strategy over the processing zone), and the direction of lines (unidirectional and bidirectional). In the unidirectional scanning strategy, the parallel lines are delivered in the same direction, whereas for the bidirectional strategy, the laser delivers each line in an opposite direction to the previous one. In the latter, the laser makes fewer movements between the lines and completes the process in a shorter time. However, the bidirectional strategy can lead to more heating than the unidirectional one, due to the lower time between the ablation of two consecutive lines. Figure 4 shows a sketch of the various laser scanning parameters and the two scanning strategies. The length of the lines is 20 mm and the width of the scan zone is kept at approximately 50 µm, varying slightly for a hatch pitch of 15 and 20 µm, giving scan widths of 45 and 40 µm, respectively. 

The crater diameter, defined as the diameter of the ablated region, measured using percussion-drilled *Stavax* with 15.4 µJ pulses, is 20 µm [3], corresponding to the largest hatch pitch in this set of experiments. The grooves are cut with wire EDM at four different positions, each 2 mm apart, before being hot-mounted, grinded, and polished for cross-sectional characterization with a digital microscope (Keyence VHX-6000, Japan). The depth and width of the grooves are measured with Keyence software. This destructive method to view the cross-sections is preferred over confocal microscopy because the high aspect ratio of some grooves leads to erroneous results with the latter measurement method.

### 3.2. Simulations

For the simulation of the grooves, a pulse-by-pulse approach is used, meaning that the ablations following every pulse are calculated separately. The simulation approach is as follows:

1. The position of the center of the pulse is calculated;
2. For each mesh point, the inclination of the surface (based on the least squares plane through the 8 mesh points around it) is calculated;
3. For each mesh point, the feature width perpendicular to the scanning direction is found, and the temperature-dependent threshold fluence and reflectivity are calculated;
4. For each mesh point, the absorbed fluence, dependent on the inclination, distance from the pulse center, feature width, and vertical distance to focus height, is calculated;
5. For each mesh point, the ablation depth parallel to the laser beam propagation direction generated by the pulse is calculated;
6. The ablation depths are subtracted from the current geometry;
7. The above steps are repeated until all pulses are calculated.

**Figure 5:**
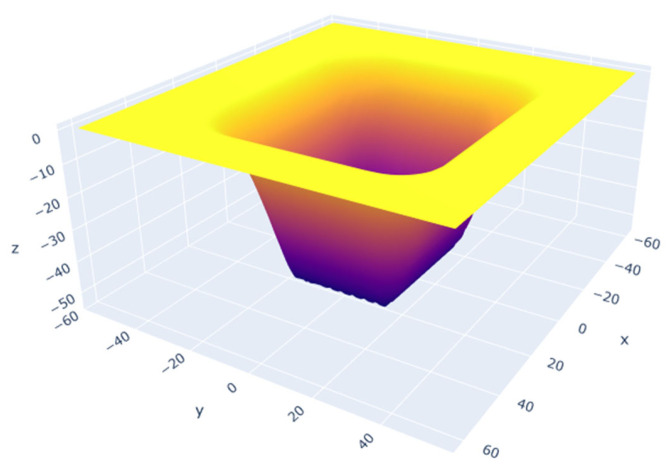
Example of a bidirectional simulation result in three dimensions. All units are in µm.

In previous studies, it has been demonstrated that all percussion-drilled cavity simulations achieve 99% convergence for a mesh resolution of 0.8 µm [3]. Therefore, this resolution is also used for the x-y mesh for all groove simulations in the present study. An example of the final groove geometry is shown in Figure 5. The following analysis focuses on the shape and dimensions of the two-dimensional sections of these grooves (read: y-z slice in the middle of the groove of Figure 5). 

## 4. Results and Discussion

### 4.1. Experiments

**Figure 6:**
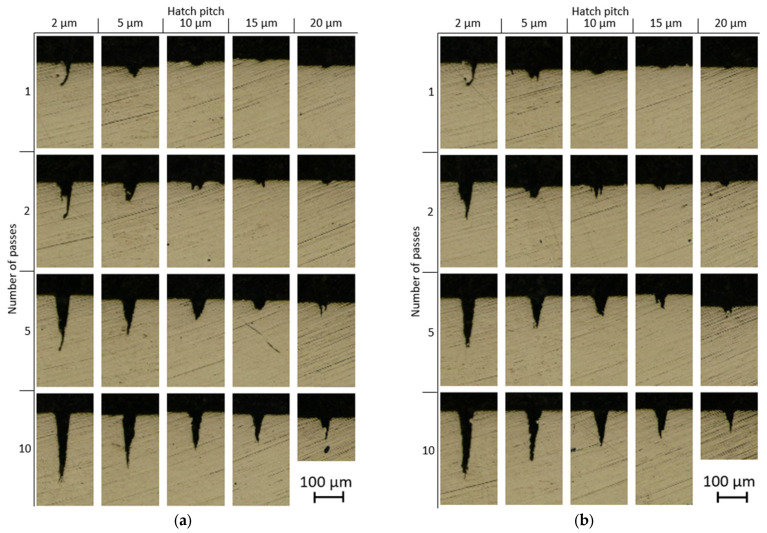
Cross-sectional 7 mm profiles from the start of the grooves of (**a**) unidirectional and (**b**) bidirectional grooves, as a function of hatch pitch and the number of passes.

A set of cross-sectional profiles of the grooves made with unidirectional and bidirectional laser strategies is shown in Figure 6a,b, respectively.

The parallel lines laser strategy generates U- or V-shaped grooves for most of the tested conditions, especially where the different scanning lines cannot be discerned, even when the hatch pitch is as large as 15 µm. Figure 6 also shows that the shape of the groove is influenced by both the number of passes and the hatch pitch. The depth increases with an increasing number of passes and a decreasing hatch pitch, while the width is not noticeably influenced by these parameters. This is expected since both a higher number of passes and a lower hatch pitch mean a higher energy density on the target material, and the width of the groove is kept almost constant by allowing the number of lines to reach a scan width of around 50 µm. 

**Figure 7:**
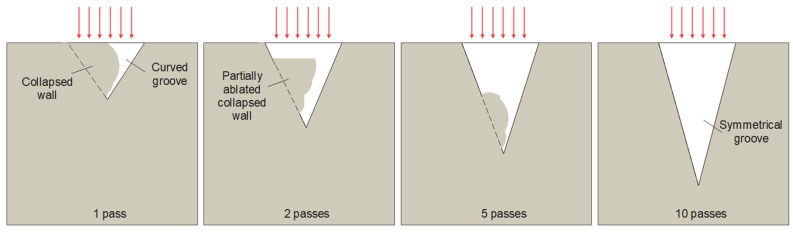
Schematical explanation of the formation of the curved groove with a 2 µm hatch pitch.

Another notable phenomenon is the curvature in the grooves with a hatch pitch of 2 µm and a low number of passes (see Figure 6a,b, for 1 pass). A similar phenomenon has also been observed and studied by Zhao et al. [16] with picosecond pulses in the titanium alloy. The bending at a high aspect ratio was attributed to the laser polarization, the laser interaction with the ejected material or plasma inside and above the crater, and the morphology on the crater surface. Contradictory to [16], in this case, the aspect ratio of the grooves is much lower and the curvature is not present anymore at higher numbers of passes. Hence, the reason for the groove curvature seems to be the re-solidification of the molten groove wall during the first pass. In other the words, the groove wall collapsed due to extreme power density at 2 µm hatch pitch in the first pass. A similar phenomenon has been documented by Zhidkov et al. [5] for the high-fluence regime, leading to melting within the heat-affected zone. The suggested sequence of events is explained schematically in Figure 7. During the first pass with a hatch pitch of 2 µm, the irradiated material surface is mostly flat, leading to high absorbed laser energy. This high energy absorption, combined with the small hatch pitch and a high number of lines, causes ablation as well as melting. This results in the wall collapsing, which gives rise to a narrow and irregular groove. The asymmetrical shape of the crater with collapsed wall can be attributed to the sequence of lines during laser scanning (up–down or down–up in Figure 4), which causes non-uniform heat distribution inside the material and asymmetrical melting. From the second pass-on, the total energy density decreases due to the reflection caused by the inclined walls. The combination of higher reflectivity and distribution of the laser fluence over a larger surface area reduces the absorbed energy density and leads to cold ablation, where the unablated material remains in a solid state. As a result, the second pass deepens the cavity and partially ablates the collapsed wall. At 10 passes, the collapsed wall is fully removed and the groove is symmetrical. 

**Figure 8:**
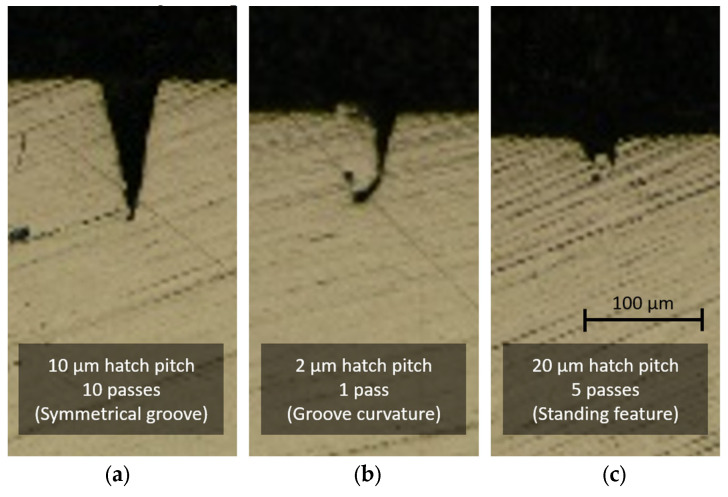
Cross-sectional profiles of (**a**) a regular groove with a hatch pitch of 10 µm and 10 passes, (**b**) an anomaly due to wall collapsing with a hatch pitch of 2 µm and 1 pass, and (**c**) an anomaly due to an overly large hatch pitch of 20 µm and 5 passes. All grooves are made with the bidirectional scanning strategy.

At large hatch pitches (15 and 20 µm), the grooves are less well-defined and traces of remaining material can be seen on several cross-sections. Figure 8 highlights the differences in groove profiles. 

The influence of the direction of the lines (unidirectional or bidirectional) on the shape of the grooves is investigated through two-sided paired T-tests based on the width and depth of the grooves. The results show that there are no differences between the two laser strategies on the depth and width of the grooves (*p* = 0.19 and *p* = 0.52, respectively).

### 4.2. Simulations without Geometry-Dependent F_th_ and R

**Figure 9:**
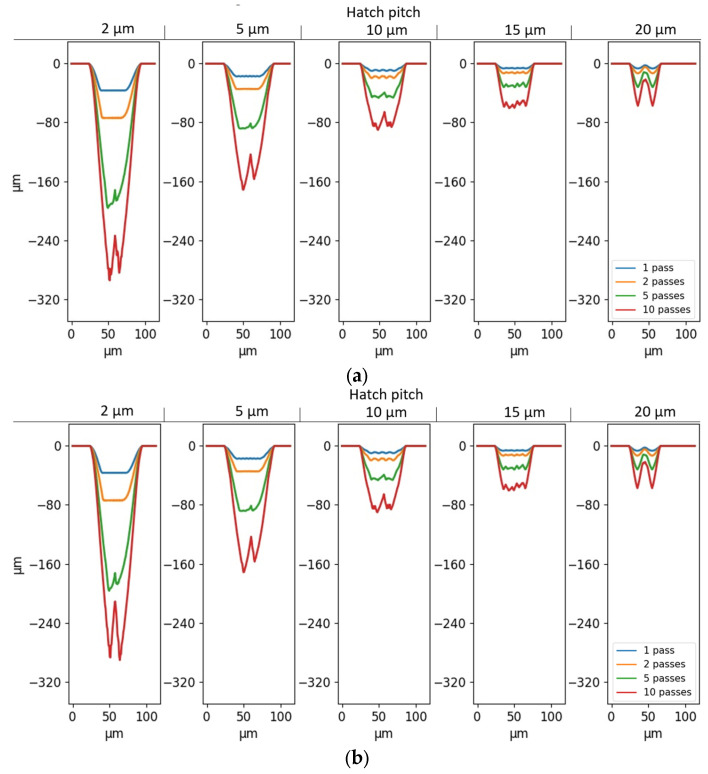
Simulated cross-sections of (**a**) unidirectional and (**b**) bidirectional grooves without geometry-dependent material parameters, as a function of hatch pitch and the number of passes.

Analogous to Figure 6, the cross-sectional profiles of the simulated grooves without geometry-dependent threshold fluence and reflectivity (i.e., with Equations (7) and (8)) are shown in Figure 9a,b. 

The simulation results for all the hatch pitches show grooves with a wavy bottom surface. Overall, the bottoms of the simulated cavities are wider than their experimental counterparts, as seen in Figure 6. Similarly to the experiments, higher numbers of passes and lower hatch pitches lead to significantly deeper but not wider grooves. Moreover, the simulations show no difference in width between the cavities created with the different direction strategies, and only minor differences in depth. Again, the two-sided paired T-test shows that there is no significant effect of line directions on the depth for simulations (*p* = 0.48). Since no laser direction effect is found in the experiments and simulations, the following investigations will be performed solely with the unidirectional laser strategy.

Compared to the experiments, the simulations consistently underestimate the depth of the grooves. In fact, the simulated grooves are, on average, 44 ± 17% shallower than the experimental ones. Moreover, the width of the grooves is consistently underestimated by the simulations, with an average error of 15 ± 11%.

### 4.3. Fitting of the Geometry-Dependent F_th_ and R

The first step towards simulating the grooves with geometry-dependent $F_{th}$ and R is to find $c_{Fth}$ and $c_{R}$ through fitting. 

**Figure 10:**
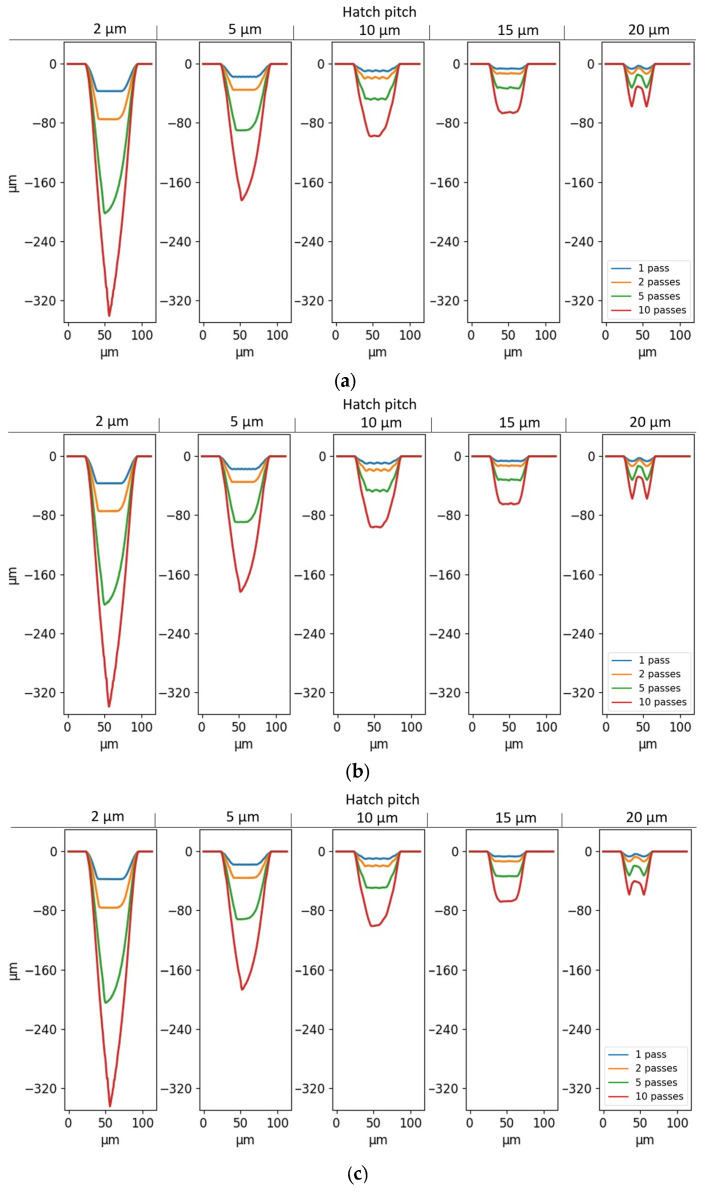
Simulated cross-sections of unidirectional grooves with geometry-dependent threshold fluence, as a function of hatch pitch and number of passes. (**a**) $c_{F_{th}}$ = 0.01; (**b**) $c_{F_{th}}$ = 0.02; (**c**) $c_{F_{th}}$ = 0.043.

The first fitting is carried out only for $c_{F_{th}}$ (i.e., with the inclusion of Equation (16)), meaning that *R* is kept independent of the feature geometry. Because the threshold fluence should always be positive, $c_{F_{th}}$ should always be smaller than $\left(F_{th}\left(T_{0}\right)\times resolution\right)$. With a resolution of 0.8 µm and $F_{th}\left(T_{0},\;\infty \right)=0.055\;J/cm²$, the upper boundary condition is $c_{F_{th}}<0.044$. The cross-sections of the simulated grooves for $c_{F_{th}}=0.01,\;0.02,$ and 0.043 are shown in Figure 10a–c, respectively.

The introduction of this geometry-dependent factor increases the smoothness of the bottom surface of the grooves, especially with larger $c_{F_{th}}$. Moreover, it can be seen that the introduction of Equation (16) in the model does not have any impact on the total depth and width of the simulated grooves. This is expected, since $c_{F_{th}}$ allows for ablation where, previously (in Section 4.2), the absorbed fluence was found to be lower than the threshold fluence. By lowering the threshold fluence in standing features, ablation can happen further away from the beam center and at higher inclinations, which results in the elimination of unablated standing features. Closer to the beam center and at low inclination angles, i.e., where the absorbed fluence is already larger than the threshold fluence, a reduction in the threshold fluence has little-to-no impact. $c_{F_{th}}=0.043$ will be used further in this study because of the smoother nature of the results for hatch pitches of 10 µm and higher.

In order to find $c_{R}$, a second fitting is performed with both Equations (16) and (17), and with $c_{F_{th}}=0.043$. In order to prevent *R* from becoming negative, Equation (17) can be rewritten as follows:

(18) $$R\left(T\right)=max\left\{R\left(T_{0}\right)-\frac{c_{R}}{width},\;0\right\}$$

**Figure 11:**
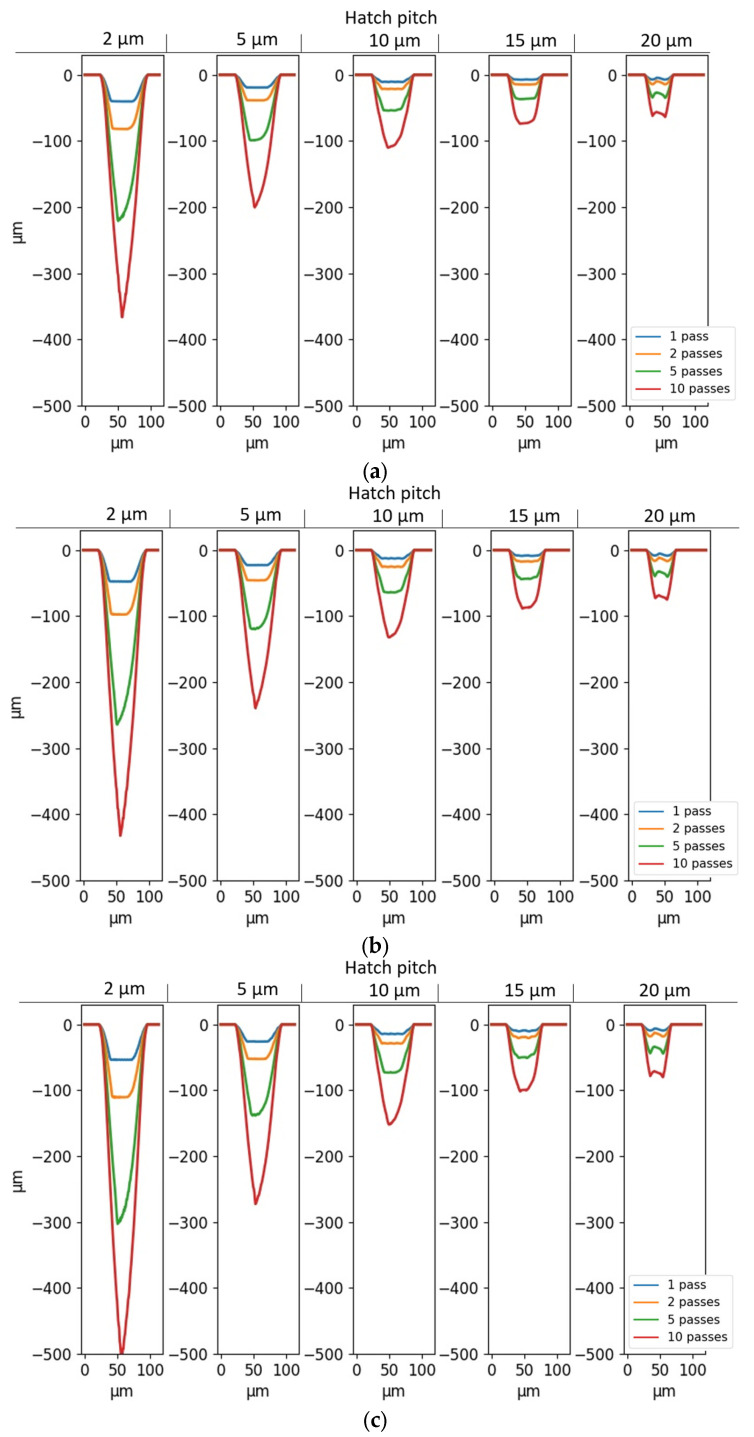
Simulated cross-sections of unidirectional grooves with geometry-dependent threshold fluence ($c_{F_{th}}$ = 0.043) and reflectivity, as a function of hatch pitch and the number of passes. (**a**) $c_{R}$ = 1; (**b**) $c_{R}$ = 5; (**c**) $c_{R}$ = 50.

Figure 11a–c show the cross-sections of the simulated grooves for $c_{R}=1,\;5,$ and 50, respectively.

**Figure 12:**
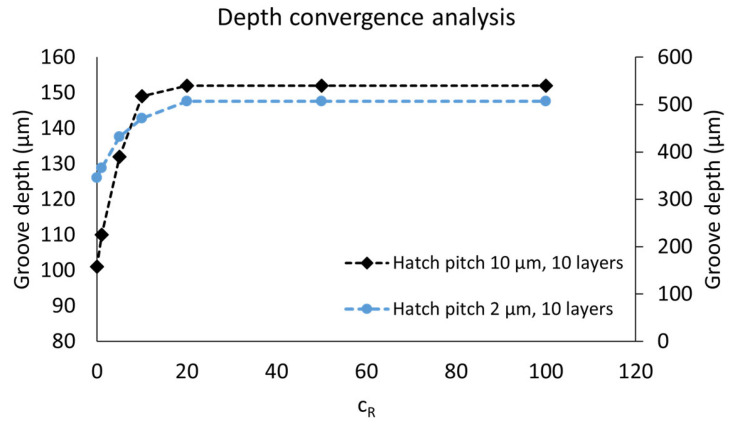
Depth convergence analysis for grooves obtained with 10 layers with hatch pitches of 2 µm (primary vertical axis) and 10 µm (secondary vertical axis).

The introduction of Equation (18) in the model has a clear influence on the depth of the grooves as well as the shape of the profiles. An increase in $c_{R}$ leads to a larger depth and a sharper profile, with better resemblance to the experimental cross-sections. However, there is a saturation between $c_{R}=5$ and $c_{R}=50$, where an increase in $c_{R}$ only has a limited effect on the simulation result. This saturation is shown in Figure 12, where the depths of simulated grooves (with hatch pitches of 2 and 10 µm and 10 layers) are plotted as a function of $c_{R}$.

### 4.4. Discussion on the Modelling Deviations

**Figure 13:**
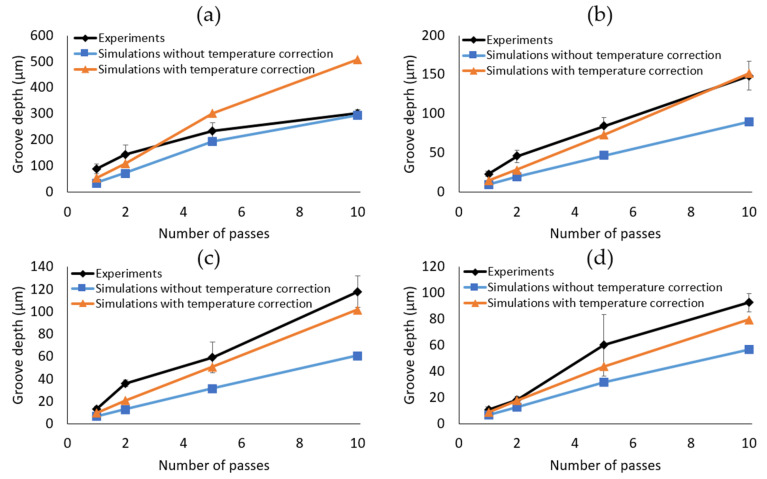
Depth comparison of the experimental and simulated grooves, with *(*$c_{F_{th}}=0.043$, $c_{R}=50$) and without geometry-dependent material parameters for different hatch pitches: (**a**) 2 µm, (**b**) 10 µm, (**c**) 15 µm, and (**d**) 20 µm in function of the number of passes. The error bars represent the standard deviations.

**Figure 14:**
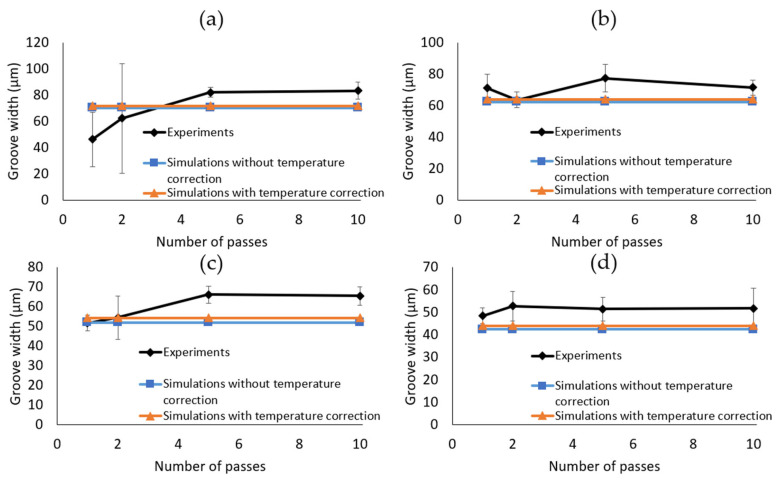
Width comparison of the experimental grooves and the simulated ones, with *(*$c_{F_{th}}=0.043$, $c_{R}=50$) and without geometry-dependent material parameters for different hatch pitches: (**a**) 2 µm, (**b**) 10 µm, (**c**) 15 µm, and (**d**) 20 µm in function of the number of passes. The error bars represent the standard deviations.

When comparing Figure 11c and Figure 6a, it is clear that the shape of the simulated profiles agrees with their experimental counterparts, with the main difference being the grooves with a 2 µm hatch pitch, for which the wall was found to collapse in the first pass. On the simulated profiles with geometry-dependent $F_{th}$ and R, the different lines are non-differentiable, except for the simulations with a 20 µm hatch pitch, which is equal to the spot size. Figure 13 and Figure 14 compare the depths and widths, respectively, of the experimental profiles with the simulated ones, both with and without geometry-dependent $F_{th}$ and R. The simulations with geometry-dependent material parameters are performed with $c_{F_{th}}=0.043$ and $c_{R}=50$.

It can be seen from Figure 13 that the depths of the simulated grooves are in good agreement with the experimental cross-sections. However, Figure 13a also shows that the depth for hatch pitch = 2 µm is largely under- and then overestimated by the simulations. This is due to the collapsed wall mentioned in Section 4.1, which cannot be modelled. With regards to the other hatch pitches (5 to 20 µm), the groove depths are, on average, still slightly underestimated by the simulations (an average error of 22 ± 14%), but the corrected model shows major improvements compared to the underestimations of the original one (an average error of 44 ± 13%). Considering the fact that the average standard deviation on the measured profile depths is 22%, the average simulation error of 22 ± 14% is justifiable, and it can be perceived that the simulations agree with the experiments. 

The average error for simulation depths without geometry-dependent material parameters for hatch pitches 5 to 20 µm and 1 pass is 50%. For 10 passes, this average error is reduced to 38%. After the inclusion of the geometry-dependent material parameters, this error decreases to 16% and 6%, respectively. This decreasing trend indicates that the model is relatively more accurate for larger numbers of passes and is significantly improved by the geometry-dependent material parameters.

With regards to the width of the grooves, Figure 14 shows that the geometry-dependent modelling parameters have a minor influence on the simulated grooves. Furthermore, in contrast to Figure 13, there are no trends between the hatch pitch or the number of passes and groove widths. The quantitative analysis, performed on hatch pitches from 5 to 20 µm, shows that the simulations underestimate the groove widths by 10 ± 7% on average. The average standard deviation on the measured profile widths is 12%, which is larger than the simulation error. Hence, the conclusion that the simulations agree with the experiments can be extended to the groove widths. 

In this corrected TTM, the incubation is only considered where ablation occurs. However, in reality, the target material also absorbs energy where the fluence is lower than the ablation threshold, causing the unablated material to heat up. This has a direct effect on the reflectivity and threshold fluence and should result in slightly wider and possibly deeper grooves than simulated in this study, which in turn can lead to better agreements with the experiments. Heinigk et al. (2021) showed that the simulation results of the TTM coupled with heat equations can be highly accurate, but they have high computational demands. Additionally, the present model does not take into account the temperature dependence of the optical penetration depth. A full characterization of the complex refractive index could be used to increase the quality and reliability of the simulation results.

## 5. Conclusions

In this work, we presented a correction and validation of the two-temperature model for the fs laser ablation of grooves. The TTM was updated with geometry-dependent material parameters to take into account the heating of the material in standing features. The temperature in standing features was found to be inversely proportional to the local width of the feature. This made it possible to link the temperature-dependent material parameters—reflectivity and threshold fluence—to the geometry of the target material without having to export the geometry to a FEM solver for temperature calculation. The model was validated for grooves with various hatch pitches, numbers of passes, and scanning directions. The results show that the corrected model matches with the experiments, both in terms of the profile shape, depth, and width for hatch pitches larger than 2 µm. The approach used in this study is highly promising for other metallic materials. However, since the relationship between the corrected parameters and the geometry is material-dependent, an experimental approach to find $c_{R}$ and $c_{F_{th}}$ should be designed. Additionally, this model could be extended to three-dimensional laser strategies, such as cross-hatching, concentric circles, and more.

## Figures and Tables

**Table 1:** Variables for ANSYS transient thermal simulations.

Feature height H [µm]	5; 10; 15; 20; 25
Feature angle [°]	10; 20; 30; 40; 50
Measurement height h [µm]	5; 10; 15; 20

**Table 2:** Material parameters for stainless steel.

$F_{th}\left(1\right)$ [4]	0.1001 J/cm²
$F_{th}(>20)$ [4]	0.055 J/cm²
κ [14]	4.49
n [14]	3.81
$\delta \left(1\right)$ [4]	32.77 nm
δ(>20) [4]	18.00 nm
S [8]	0.8

**Table 3:** An overview of the laser parameters.

Pulse energy	15.4 µJ
Repetition rate	500,000 Hz
Scanning speed	100 mm/s
Scan width	~50 µm
Number of passes	1; 2; 5; 10
Hatch pitch	2; 5; 10; 15; 20 µm
Direction	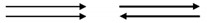 (unidirectional) (bidirectional)

## Data Availability

The datasets generated and/or analyzed during the current study are available from the corresponding author on reasonable request.

## References

[1] Leitz K.-H., Redlingshöfer B., Reg Y., Otto A., Schmidt M. (2011). Metal Ablation with Short and Ultrashort Laser Pulses. Phys. Procedia.

[2] Chichkov B.N., Momma C., Nolte S., Alvensleben F., Tünnermann A. (1996). Femtosecond, picosecond and nanosecond laser ablation of solids. Appl. Phys. A.

[3] Vanwersch P., Schildermans S., Nagarajan B., Van Bael A., Castagne S. (2022). Three-Dimensional Modelling of Femtosecond Laser Ablation of Metals. Lasers Manuf. Mater. Process..

[4] Audouard E., Mottay E. (2016). Engineering model for ultrafast laser microprocessing. Front. Ultrafast Opt. Biomed. Sci. Ind. Appl. XVI.

[5] Zhidkov M.V., Vershinina T.N., Golosova O.A., Kudryashov S.I., Ionin A.A. (2020). Surface texturing of steel by femtosecond laser and accompanying structure/phase transformations. Opt. Laser Technol..

[6] Jee Y., Becker M.F., Walser R.M. (1988). Laser-induced damage on single-crystal metal surfaces. JOSA B.

[7] Neuenschwander B., Jaeggi B., Schmid M., Dommann A., Neels A., Bandi T., Hennig G. (2013). Factors controlling the incubation in the application of ps laser pulses on copper and iron surfaces. Laser Applications in Microelectronic and Optoelectronic Manufacturing (LAMOM) XVIII.

[8] Di Niso F., Gaudiuso C., Sibillano T., Mezzapesa F.P., Ancona A., Lugarà P.M. (2014). Role of heat accumulation on the incubation effect in multi-shot laser ablation of stainless steel at high repetition rates. Opt. Express.

[9] Ahmmed K.M.T., Grambow C., Kietzig A.-M. (2014). Fabrication of Micro/Nano Structures on Metals by Femtosecond Laser Micromachining. Micromachines.

[10] Cangueiro L., Audouard E., Martin P.E., Mottay E., Ramos-de-Campos J.A., Kupisiewicz A., Bruneel D. (2018). Model Ultrafast Laser Micromachining. Laser-Based Micro-Nanoprocessing XII.

[11] Heinigk C., Barthels T., Nießen M., Schulz W. (2021). A multi-scale model for ultra short pulsed parallel laser structuring—Part I. Micro-Scale Model JLMN.

[12] Paquin R.A., van Stryland E.W., Optical Society of America (1995). Properties of Metals in Handbook of Optics: Devices, Measurements, and Properties.

[13] Ward M.R. (1971). Electrical Engineering Science.

[14] Steen W.M., Mazumder J. (2010). Laser Material Processing.

[15] Han J., Malek O., Vleugels J., Braem A., Castagne S. (2022). Ultrashort pulsed laser ablation of zirconia-alumina composites for implant applications. J. Mater. Process. Technol..

[16] Zhao W., Wang L., Yu Z., Chen J., Yang J. (2019). A processing technology of grooves by picosecond ultrashort pulse laser in Ni alloy: Enhancing efficiency and quality. Opt. Laser Technol..

